# A Transcription Factor Code Defines Nine Sensory Interneuron Subtypes in the Mechanosensory Area of the Spinal Cord

**DOI:** 10.1371/journal.pone.0077928

**Published:** 2013-11-04

**Authors:** Marta Garcia Del Barrio, Steeve Bourane, Katja Grossmann, Roland Schüle, Stefan Britsch, Dennis D.M. O’Leary, Martyn Goulding

**Affiliations:** 1 Molecular Neurobiology Laboratory, The Salk Institute for Biological Studies, La Jolla, California, United States of America; 2 Urologische Klinik/Frauenklinik und Zentrale Klinische Forschung, Klinikum der Universität Freiburg, Freiburg, Germany; 3 Department of Medical Genetics, Max-Delbrück-Center for Molecular Medicine, Berlin-Buch, Germany; 4 Institute for Molecular and Cellular Anatomy Ulm University, Ulm, Germany; University of Cincinnatti, United States of America

## Abstract

Interneurons in the dorsal spinal cord process and relay innocuous and nociceptive somatosensory information from cutaneous receptors that sense touch, temperature and pain. These neurons display a well-defined organization with respect to their afferent innervation. Nociceptive afferents innervate lamina I and II, while cutaneous mechanosensory afferents primarily innervate sensory interneurons that are located in lamina III–IV. In this study, we outline a combinatorial transcription factor code that defines nine different inhibitory and excitatory interneuron populations in laminae III–IV of the postnatal cord. This transcription factor code reveals a high degree of molecular diversity in the neurons that make up laminae III–IV, and it lays the foundation for systematically analyzing and manipulating these different neuronal populations to assess their function. In addition, we find that many of the transcription factors that are expressed in the dorsal spinal cord at early postnatal times continue to be expressed in the adult, raising questions about their function in mature neurons and opening the door to their genetic manipulation in adult animals.

## Introduction

Interneurons in the dorsal spinal cord receive and process multiple types of cutaneous sensory information, including pain, temperature, pressure and vibration [1]–[7]. In addition to relaying cutaneous stimuli, interneurons in the dorsal horn transmit propioceptive information from Group II and III muscle afferents [8]. These cutaneous sensory afferents terminate in the dorsal horn in a modality-specific manner [1], [2], [7]. Nociceptive information is received primarily in lamina I–II from two different classes of sensory afferent neurons that are distinguished molecularly as peptidergic (CGRP^+^/TrkA^+^) C/Aδ fiber afferents and non-peptidergic (Mrgprd^+^/IB4^+^/Ret^+^) C fiber afferents [7]. There are also nociceptive Aδ fibers that terminate in lamina I [7]. Low threshold mechanoreceptors (LTMRs) that transduce innocuous cutaneous mechanosensory information innervate first order sensory interneurons that are located between inner lamina II (IIi) and lamina IV [8]. As a general rule, C-fiber LTMRs primarily project to lamina II, Aδ-fiber LTMRs project to laminae IIi and III, while Aβ-fiber RA-LTMRs project mainly to laminae III–IV [7], [8]. Proprioceptive information in the dorsal spinal cord is mainly processed by neurons in laminae IV–VI, although many proprioceptors project to more ventral regions of the spinal cord where they innervate premotor interneurons and motor neurons [1], [9]–[11].

Despite the importance of the dorsal spinal cord for the reception and transduction of cutaneous mechanosensory stimuli, we know very little about the neuronal composition of the central circuits that gate and transmit this information. Efforts to probe the organization of these circuits have been hampered by their complexity, and by an inability to molecularly define discrete populations of sensory neurons and ascribe functions to them. Recently, a number of developmentally-regulated transcription factors that are expressed in the developing dorsal horn have been identified [11]–[16] that provide an entry point for identifying the sensory interneuron cell types that play essential roles in processing and transducing cutaneous somatosensory information. Using a battery of transcription factors that are expressed at late embryological and early postnatal stages, we have begun to probe the molecular diversity of interneurons in laminae III–IV, which is primarily innervated by cutaneous mechanoreceptors. The resultant analysis of multiple transcription factors in combination with Pax2, Gbx1 and Lmx1b, which are more broadly expressed in the dorsal spinal cord, has allowed us to identify nine molecularly-distinct interneuron populations in lamina III–IV at postnatal and adult stages. More importantly, the systematic identification of nine molecularly-defined sensory interneuron cell types in lamina III–IV has set the stage for functionally dissecting mechanosensory circuits in lamina III–IV using genetic and molecular approaches similar to those employed for studying central pattern generator (CPG) networks (for recent reviews see [17]–[19]). Consequently, we can now: 1) examine the role that specific neural populations play in transducing the sensation of touch, 2) determine the contribution that cutaneous stimuli make to the dynamic control of movement, and 3) further our understanding of how somatosensory information is coded by spinal cord interneurons.

## Materials and Methods

### Animals

All protocols for animal experiments were approved by the IACUC of the Salk Institute for Biological Studies and follow the NIH guidelines for animal use. The mouse lines used in this study have been described previously: *Pax2-Cre* [20]; *R26* ^floxstop-Tomato^ (*Ai14*) [21]; *MafB-GFP* [22]; *GAD67-GFP* [23]; *Lmx1b* knockout [24]; *RORα-IRES-Cre* [25]. All mice were genotyped by PCR using allele-specific primers for each strain. For timed pregnancies, midday on the day of the vaginal plug was designated as embryonic day (E) 0.5. The day of birth was designated as P0.

### Tissue Preparation and Immunohistochemistry

P0-adult mice were euthanized and perfused with 4% paraformaldehyde in PBS (PF). Their spinal cords were then post-fixed for 30–60 mins in 4% PF at 4°C (P0) or at room temperature (adult). Spinal cords were rinsed and cryoprotected in 20% sucrose in PBS (4°C) prior to embedding in OCT (Tissue-Tek). Immunostaining of frozen spinal sections was performed by incubating 20 µm thick sections with primary antibodies, which were then detected using species-specific secondary antibodies conjugated with Cy2, Cy3 and Cy5 (Jackson Laboratories) or FITC (Invitrogen). Three-color images were captured using either an Axioskop 2 Mot Plus microscope or a Zeiss LSM510 Laser Scanning Confocal Microscope. AxioVision and Adobe Photoshop software was used for image analysis, data processing and presentation.

### Antibodies

The following commercially available antibodies were used: monoclonal anti-Lhx1 and anti-Lhx5 (4F2-10: Developmental Hybridoma Studies Bank); polyclonal anti-Pax2 (71–6000, Zymed; 1∶1000); chick anti-GFP (GFP-1020, Aves lab; 1∶1000); rabbit anti-GFP (A-11122, Molecular Probes; 1∶1000); chick anti-β-galactosidase Abcam; 1∶1000), guinea pig anti-VGluT1 (AB5905, Millipore; 1∶2000); goat anti-MafB (SC10022, Santa Cruz Biotechnology; 1∶500); rabbit anti-MafA (Bethyl Laboratories; 1∶750); rabbit anti-c-Maf (Bethyl Laboratories; 1∶2500); guinea pig anti-Lbx1 ([26]; 1∶1000. Rabbit and guinea pig anti-Gbx1 antibodies were generated against aa 61–308 of the Gbx1 protein (used at 1∶10,000). Rabbit anti-MafB antisera provided by Carmen Birchmeier and Thomas Müller (Max-Delbrück-Center for Molecular Medicine, Berlin, Germany) was used at 1∶500. Anti-Lmx1b antisera provided by Thomas Jessell (Columbia University, USA) was used at 1∶1000. A rabbit anti-RORβ antibody that recognizes the ligand-binding domain (LBD) of RORβ was generated and affinity purified using immobilized GST-rat RORβ LBD then used at 1∶100.

## Results

### Identification of Transcription Factors Expressed in the Mechanosensory Area of the Dorsal Horn at Postnatal and Adult Stages

**Figure 1:**
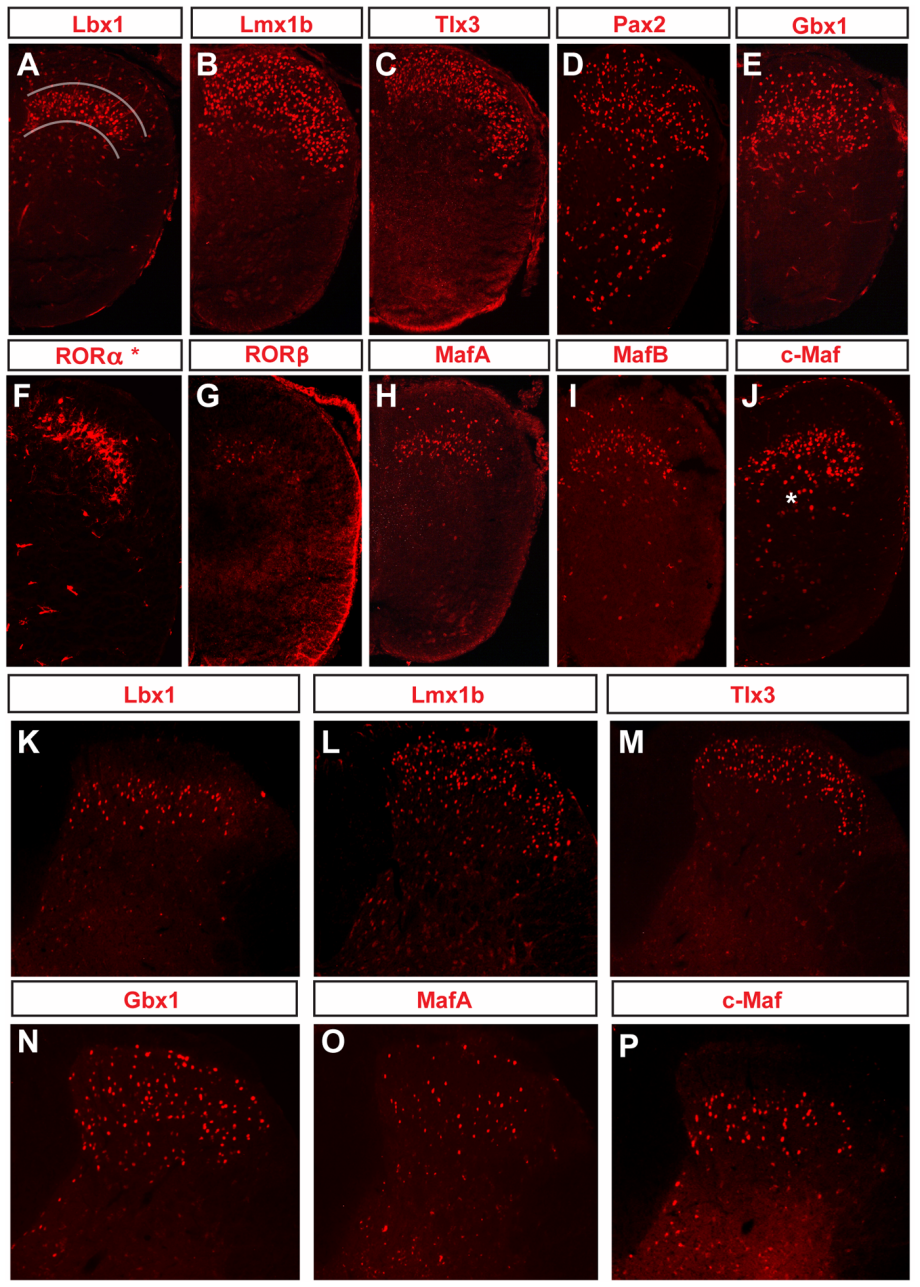
Expression of Lbx1, MafA, c-Maf, RORα, RORβ, Pax2, Lmx1b, Gbx1 and Tlx3 in the postnatal and adult spinal cord. Immunostaining of trancription factors that are enriched in the dorsal horn at P4 (A–J) and adult (K–P) stages. Lbx1, Lmx1b, Tlx3, Gbx1, MafA and c-Maf expression is maintained in the spinal cords of five month old mice (K–P). RORα* denotes *RORα-Cre; R26* ^floxstop-GFP^ in all figures. The lines in A indicate the border of lamina III–IV.

We used a battery of antibodies, in combination with previously characterized mouse reporter lines, to map the expression of multiple transcription factors in the postnatal dorsal horn and test whether they are expressed in discrete populations of laminae III–IV sensory interneurons. This initial survey identified a number of transcription factors with known roles in neuronal specification and differentiation, all of which are expressed in the dorsal spinal cord at late embryonic and postnatal stages (Fig. 1; data not shown). We focused our analysis on thoracic and lumbar spinal cord levels, as there was no discernable difference in the expression patterns of these transcription factors along the anterior-posterior (A–P) axis at lower spinal cord levels. Transcription factor expression was analyzed at the following ages: P0, P3, P4, P7, P10, P27, and in the adult.

Two broad sets of transcription factors were identified. The first, which includes Lbx1, Lmx1b, Tlx3, Pax2 and Gbx1, is comprised of transcription factors that display relatively broad patterns of expression in the dorsal horn (Fig. 1A–E). During embryogenesis, these transcription factors are expressed broadly in the developing spinal cord [11], [12], [14], [15], [27], [28], whereas at postnatal times, we find that they are enriched in the dorsal horn, including laminae III–IV (Fig. 1K–N). The second set of transcription factors that we identified displays a more restricted pattern of expression in dorsal sensory interneurons. This group includes the nuclear orphan receptors RORα and RORβ, and the large Maf proteins, MafA, MafB and c-Maf. All five transcription factors displayed cell type-specific expression in the dorsal horn at postnatal stages (Fig. 1F–J, O–P), with the expression patterns of MafA, c-Maf and Gbx1 in the adult spinal cord closely resembling those seen at earlier developmental times [29], [30].

MafA, MafB and c-Maf, together with Lbx1, are all expressed in laminae III–IV at postnatal and adult stages (Fig. 1). During embryogenesis, these four transcription factors are also transiently expressed in intermediate and ventral areas of the spinal cord, where they mark subsets of ventral commissural neurons and Renshaw cells (Fig. S1; [11], [12], [31], [32]). At postnatal and adult stages, cells expressing Lbx1, MafA, MafB and c-Maf are largely restricted to laminae III–IV, with only a few neurons present in other laminae (Fig. 1A, H–J, O–P, data not shown). In the case of MafA and MafB, we detected sparse labeling of interneurons in the superficial dorsal horn (Fig. 1H, I, O), whereas c-Maf is expressed in a small number of cells in lamina V (Fig.1J, asterisk). RORβ, on the other hand, displays a more restricted pattern of expression throughout development (data not shown). In the postnatal and adult cord, RORβ is restricted to laminae III–IV, with the exception of a few cells that are located in lamina I (Fig. 1G; Fig. S2).

Lmx1b is expressed at high levels in laminae I–III, with lower levels of expression in lamina IV (Fig. 1L). Tlx3 is principally expressed in laminae I–II, although we did detect Tlx3^+^ cells in laminae III (Fig. 1J, M). Pax2-expressing cells were more broadly distributed within the dorsal horn (Fig. 1D), but were present in lower numbers in the ventral horn. The ventral Pax2^+^ cells, together with the dorsal Pax2^+^ cells, are likely to be inhibitory interneurons, due to their expression of multiple inhibitory neuron markers at early embryonic times [17], [27]–[29].

When VGluT1 immunostaining, which marks myelinated mechanosensory afferents that terminate throughout laminae IIi-V [33]–[35], was used to determine the lamina location of the cells expressing Lbx1, Gbx1, RORb, MafA and c-Maf, the interneurons expressing Lbx1 and MafA were seen to be primarily restricted to lamina III–IV (Fig. S2). Neurons that express Gbx1, RORβ and c-Maf also displayed extensive co-localization with VGluT1^+^ sensory afferents in lamina III and IV (Fig. S2).

In summary, we have identified a cohort of transcription factors that are expressed in sensory interneurons within lamina III–IV, which is the primary recipient region for innocuous mechanosensory afferents. In view of the demonstrated roles that these transcription factors play in regulating neuronal cell specification, differentiation and cell physiology [11], [12], [14], [15], [36]–[43], it is highly likely that they have important roles in controlling the physiology of dorsal sensory interneurons gating cutaneous mechanosensory stimuli.

### Lbx1, RORβ, RORα, MafB and c-Maf are Expressed by Mixed Populations of Inhibitory and Excitatory Neurons, whereas MafA is Restricted to Excitatory Neurons

**Figure 2:**
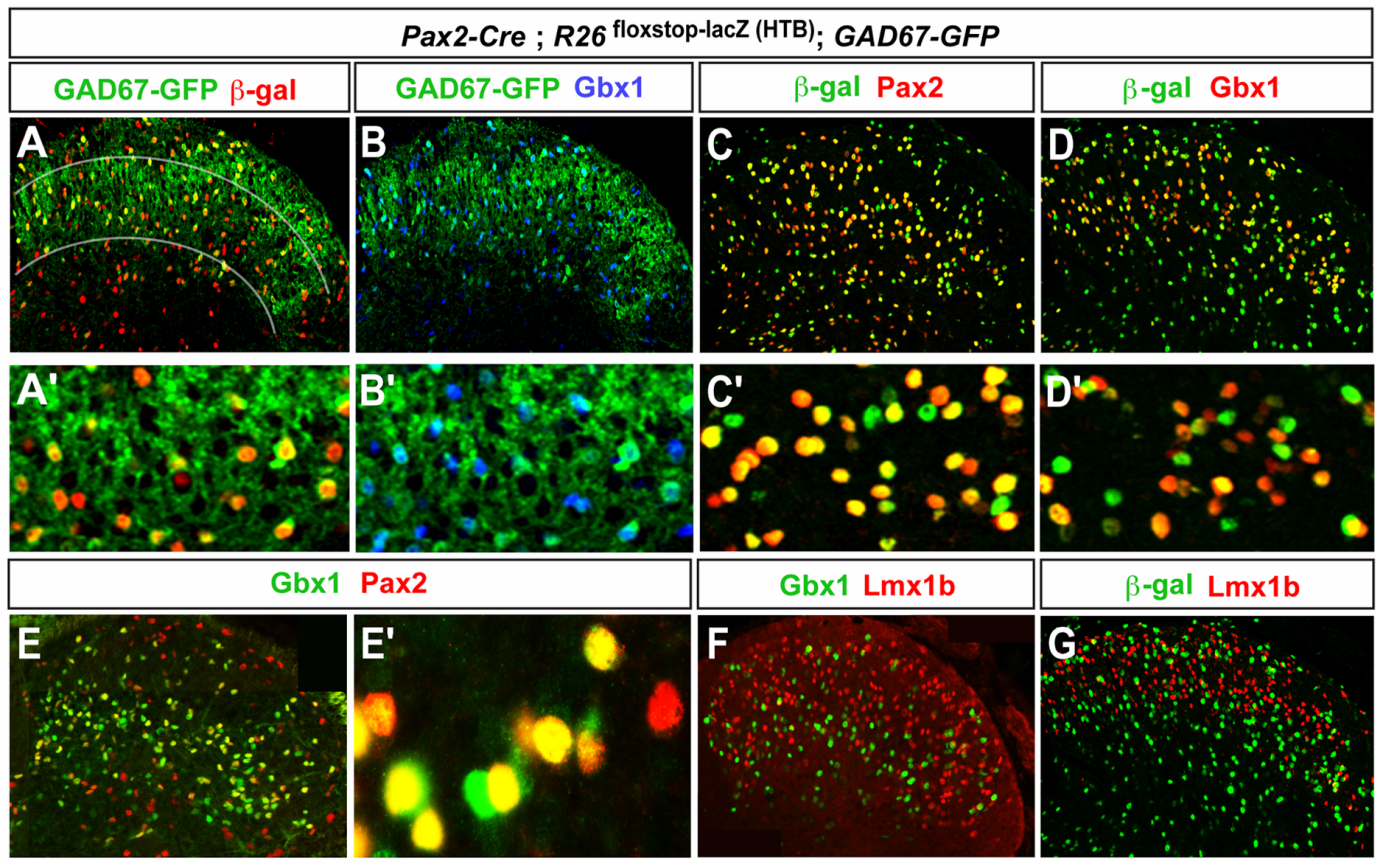
Lmx1b and Pax2 are postnatal markers of excitatory and inhibitory neurons. Analysis of Gbx1, Pax2 and Lmx1b expression in *Pax2-Cre; R26* ^floxstop-lacZ^ mice at P10 (A–D′,G) and P1 (E–F). The Pax2-derived β-gal reporter is localized to GAD67-GFP^+^ neurons in the dorsal horn (A,A′). A large fraction of the Pax2-derived β-gal cells express the Pax2 transcription factor (C,C′). GAD67-GFP and Pax2^+^ cells express the Gbx1 transcription factor (B,B′,E,E′). Lmx1b is not expressed in Gbx1^+^ neurons (F) or Pax2-derived neurons in the *Pax2-Cre; R26* ^floxstop-lacZ^ spinal cord (G). The lines in A indicate the border of lamina III–IV.

**Figure 3:**
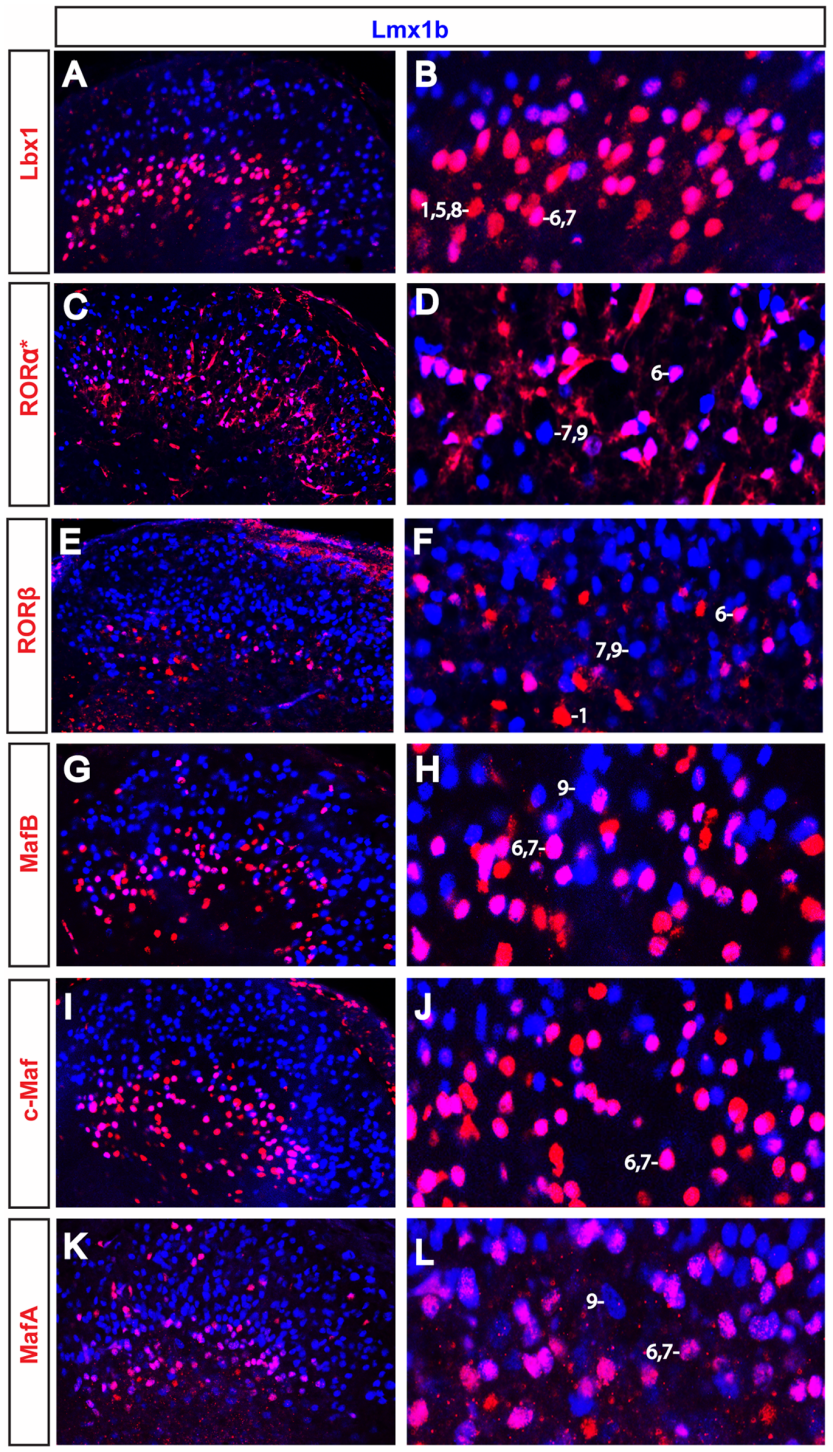
Comparative expression of transcription factors that are co-localized with the excitatory marker Lmx1b. Lbx1, RORα, RORβ, MafA, MafB and c-Maf all show overlapping expression with Lmx1b in excitatory neurons, albeit at low levels in some neurons. Analyses were performed at P0 (C–D, K–L), P7 (I–J, G–H), P8 (E–F) and P10 (A–B). Neurons were assigned a cell type number (1–9) according to their expression profile, with profiles 1–5 being classified as inhibitory neurons, while profiles 6–9 are excitatory neurons (see Table 2).

Lbx1, MafA, RORβ, MafB, c-Maf, RORα, Pax2, Lmx1b and Gbx1 continue to be expressed in laminae III–IV at late postnatal stages. As a first step toward defining the phenotype of the neurons that express these transcription factors, we asked which of these proteins are expressed in inhibitory or excitatory sensory interneurons. Previous studies have shown that Lmx1b is a marker of dIL_B_ excitatory neurons in the embryonic spinal cord, while Pax2 and Gbx1 mark inhibitory dIL_A_ inhibitory neurons [11], [12], [28], [29]. To address this question, we used glutamic acid decarboxylase 67–green fluorescence protein (*GAD67-GFP*) knock-in mice [23] to mark and trace inhibitory interneurons. Lmx1b was not detected in neurons that express GFP at any postnatal time analyzed, indicating the Lmx1b cells are not GABAergic inhibitory interneurons (Table 1, data not shown). When we analyzed *Pax2-Cre*; *R26* ^floxstop-lacZ^; *Gad67-GFP* mice, most, if not all, Pax2-derived β-galactosidase^+^ (β-gal^+^) cells in laminae III–IV expressed GFP, demonstrating that they are indeed inhibitory neurons (Fig. 2A,A′). Gbx1 also showed strong co-localization with the GAD67-GFP reporter (Fig. 2B,B′), and a large number of these Gbx1^+^ cells expressed β-gal (Fig. 3D–E′). We also detected Pax2^+^ and Gbx1^+^ cells that do not express GFP in the postnatal cord (Fig. 2A–B′). The presence of these GAD67-GFP-negative cells in the postnatal cord most likely reflects the down-regulation of GAD67 at postnatal times, which has been noted in other studies [23], [28]. Conversely, there are GAD67-GFP^+^ neurons at in the postnatal cord that do not express β-gal or Gbx1 (Fig. 2A,B). In *Pax2-Cre*; *R26* ^floxstop-lacZ^ mice, the neurons that continue to express Pax2 were found to represent only subset of the Pax2-Cre^+^ (β-gal^+^) cells in dorsal horn (Fig. 2C,C′). This is again due to the down-regulation of Pax2 (and Gbx1) in the postnatal dorsal horn [28], [30], since at earlier times Pax2 is expressed in all Pax2-Cre marked neurons (Fig. S3).

**Table 1:** Summary of the expression analyses used to define the inhibitory or excitatory phneotype of postnatal neurons that express Pax2, Gbx1, Lmx1b, RORβ, RORα, Lbx1, MafA, MafB and c-Maf.

	Pax2	Gbx1	Lmx1b	RORα	RORβ	Lbx1	MafA	MafB	c-Maf
***Gad67-GFP***	82±3%	77±4%	<1%	nd	32±2%	11±2%	<1%	13±2%	18±2%
***Pax2-Cre; lacZ***	96±2%	95±3%	<1%	nd	54±2%	18±3%	nd	32±2%	29±3%
**Gbx1**	81±4%	100%*	<1%	3±2%	51±3%	21±4%	<1%	43±3%	19±3%
**Pax2**	100%*	87±2%	<1%	nd	nd	nd	nd	nd	nd
**Lmx1b**	<1%	<1%	100%*	92±3%	48±3%	79±3	93±2	47±3%	62±4%

Pax2 and Gbx1 are inhibitory markers, whereas Lmx1b and MafA are excitatory markers. Lbx1 and RORα predominantly label excitatory neurons, as well as a small number of inhibitory neurons. MafB and c-Maf are expressed by mixed populations of inhibitory and excitatory neurons. Data is expressed as mean±s.d. RORα expression was analyzed using a *RORα* ^Cre^; *R26* ^floxstop-Tomato^ reporter. Asterisk indicates 100% by definition.

The *Pax2-Cre; R26* ^floxstop-lacZ^ reporter mouse was then used to assess whether Lmx1b co-localizes with GFP in Pax2-derived “inhibitory” neurons. No overlap between Lmx1b and β-gal expression was noted at P1 (Fig. 2G), which is consistent with our observation that Lmx1b is excluded from cells that belong to the Pax2 (dIL_A_) lineage (Fig. S3). This finding demonstrates that Pax2-Cre-derived neurons do not express the excitatory marker Lmx1b, and are thus unlikely to be glutamatergic interneurons. We also confirmed that Lmx1b does not co-localize with Gbx1 (Fig. 2F), indicating that Lmx1b and Gbx1 mark two separate cell populations in the dorsal horn. The majority of laminae III–IV cells that express either Pax2 or Gbx1, express both factors, although there are a small number of cells that express Pax2 and Gbx1 alone (Fig. 2E). Taken together, our data demonstrate that Lmx1b and Pax2/Gbx1 are specific postnatal markers of excitatory and inhibitory neurons, respectively.

**Figure 4:**
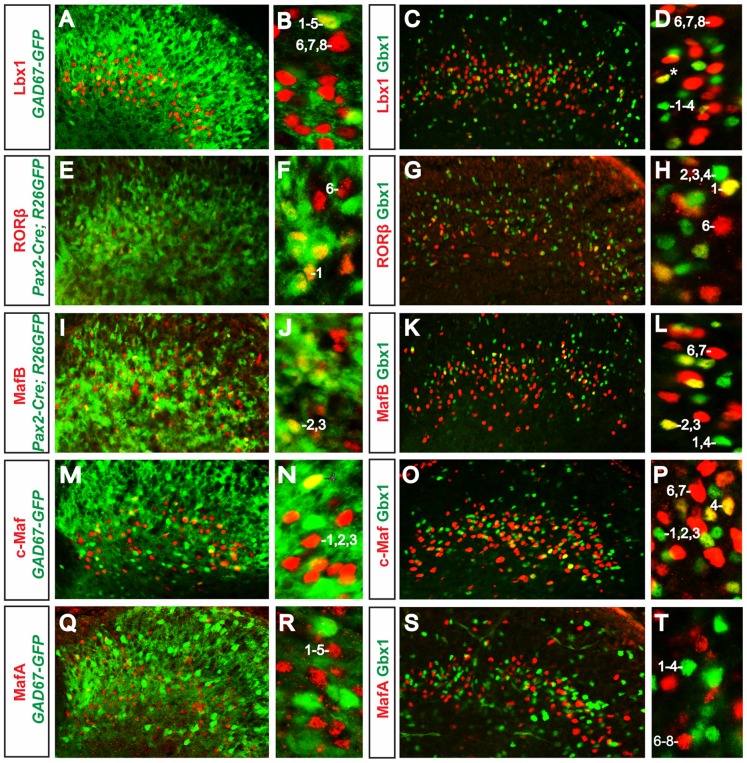
Comparison of transcription factor expression with inhibitory neuronal markers. Lbx1 (A–D), RORβ (E–H), MafB (I–L) and c-Maf (M–P) are expressed in many neurons that express the inhibitory markers Gbx1 (C–D, G–H, K–L, O–P, V–W), *GAD67-GFP* (A–B, M–N) and *Pax2-Cre; R26* ^floxstop-GFP^ (E–F, I–J). In *RORα-Cre; R26* ^floxstop-Tomat*o*^ mice (V–W), we observed a few Tomato^+^ neurons that expressed Gbx1. MafA is the only transcription factor that did not co-localize with Gbx1 (S–T) or GFP in *GAD67-GFP* and *Pax2-Cre; R26* ^floxstop-GFP^ mice (Q-R, data not shown). *Pax2-Cre; R26GFP* denotes GFP^+^ cells in *Pax2-Cre; R26* ^floxstop-GFP^mice. Spinal cords were analyzed at P0 (E–F), P1 (C–D, I–J, S–T), P2 (G–H), P7 (A–B, M–N, Q–R), P8 (V–W), and P10 (K–L, O–P). Examples of the nine different cell types (numbered 1–9) are shown. See Table 2 for further details.

We then analyzed *GAD67-GFP* mice at P3, P7 and P10 and *Pax2-Cre; R26* ^floxstop-GFP^ mice at P1 to determine whether Lbx1, RORβ, RORα, MafA, MafB and c-Maf are expressed in GABAergic inhibitory neurons (Figs. 3 and 4, summarized in Table 1). These analyses revealed that Lbx1, RORα, RORβ, MafB and c-Maf are present in mixed populations of inhibitory and excitatory neurons. At postnatal times, Lbx1 and RORα are predominantly expressed in Lmx1b^+^ excitatory neurons (Fig. 3A–D), although a small, but significant fraction of the Lbx1^+^ interneurons in *GAD67-GFP* mice are GFP^+^ (Fig. 4A–B; data not shown). This expression of Lbx1 in inhibitory neurons was confirmed by double immunostaining experiments with antibodies to Gbx1 and Lbx1 (Fig. 4D, asterisk).

Interestingly, the RORβ, MafB, and c-Maf populations appear to be more heterogeneous with respect to their neurotransmitter phenotype. Approximately 50% of the MafB and RORβ neurons express Lmx1b, and are thus excitatory. In the case of the c-Maf neurons, greater than 60% of these cells express Lmx1b (Fig. 3E–F, G–H, I–J, respectively). Conversely, approximately 50% of the RORβ^+^ cells and 30% of the MafB^+^ and c-Maf^+^ cells show co-localization with GFP in P1 *Pax2-Cre; R26* ^floxstop-GFP^ mice (Fig. 4E–F, I–J, respectively and data not shown). Likewise, dual immunostaining with Gbx1 showed ∼50%, ∼40% and ∼20% co-localization with RORβ, MafB and c-Maf, respectively (Fig. 4G–H, K–L, O–P). In the *GAD67-GFP* mice we found fewer GFP^+^/MafB^+^ and GFP^+^/c-Maf^+^ cells (∼10% and ∼20%, respectively; Fig. 4M–N; data not shown), which is probably due to the down-regulation of GAD67 in the postnatal spinal cord [28].

Our results suggest that most, if not, all of the MafA^+^ neurons in lamina III and lamina IV are excitatory glutamatergic neurons, as more than 90% of these MafA^+^ cells co-express Lmx1b (Fig. 3K–L, Table 1). Furthermore, MafA does not co-localize with GFP in *GAD67-GFP* mice at P3, P7 and P10 (Fig. 4Q–R), nor is it expressed together with Gbx1 at these times (Fig. 4S–T). In summary, Lbx1, RORβ, MafB and c-Maf are all expressed in mixed populations of inhibitory and excitatory neurons, whereas MafA is specific to excitatory neurons.

### Nine New Populations of Neurons Identified in the Mechanosensory Area by the Combinatorial Expression of the Lbx1, RORβ, RORα, MafA, MafB, c-Maf, Gbx1, Pax2 and Lmx1b Transcription Factors

**Figure 5:**
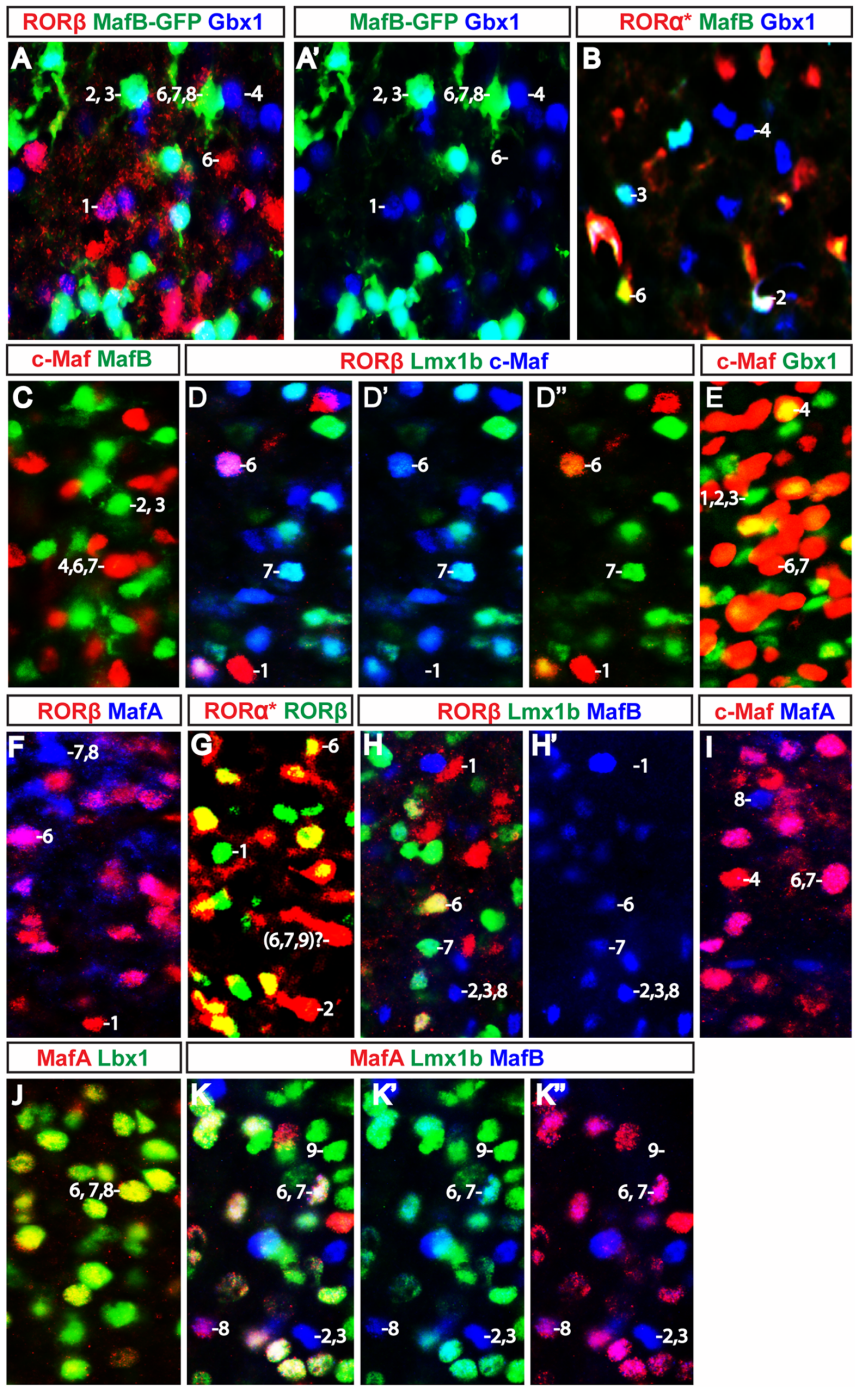
Relative expression of the transcription factors that define the transcription factor code for the different neuron populations. The RORβ inhibitory neurons that expressed Gbx1 did not express GFP in *MafB-GFP* mice but not all Gbx1-positive neurons expressed GFP (A–A′). Therefore RORβ/Gbx1 double-positive neurons do not express MafB and not all Gbx1 neurons express MafB. Tomato-positive neurons that expressed Gbx1 in *RORα-Cre; R26* ^floxstop-Tomato^ mice also expressed MafB (B), therefore all RORα^+^/Gbx1^+^ cells express MafB. No c-Maf-positive neurons expressed GFP in *MafB-GFP* mice (C) and consequently, c-Maf and MafB do not co-localize in MafB inhibitory neurons. All RORβ-positive neurons that expressed Lmx1b also expressed c-Maf (D–D”). Many c-Maf-positive neurons expressed Gbx1 (E). Many RORβ-positive neurons expressed MafA, as MafA is an excitatory marker (F). Many RORβ-positive neurons expressed Tomato in *RORα-Cre; R26* ^floxstop-Tomato^ mice (G). RORβ^+^ neurons that express MafB are Lmx1b^+^ excitatory neurons (H–H′). Most MafA expressed C-Maf although there was a significant number of cells that only expressed MafA single or c-Maf single-positive (I). All MafA-positive neurons expressed Lbx1, although some of them at very low levels like (J). All MafB-positive neurons that expressed Lmx1b also expressed MafA (K–K”). See Table 2 for number designations.

The combinatorial expression of Lbx1, RORβ, RORα, MafA, MafB, c-Maf, Gbx1, Pax2 and Lmx1b reveals the presence of at least nine different populations of neurons in laminae III–IV. These are summarized in Table 2. We have numbered each population, examples of which are shown in Figures 2–5. Within laminae III–IV, we were able to distinguish five molecularly-distinct populations of inhibitory neurons and four populations of excitatory neurons (Table 2, columns 1–5).

**Table 2:** A combinatorial transcription factor code defines nine different postnatal populations of interneurons in laminae III–IV.

	1	2	3	4	5	6	7	8	9
*GAD67-GFP*	+	+	+	+	+	–	–	–	–
Gbx1	+	+	+	+	–	–	–	–	–
Lmx1b	–	–	–	–	–	+	+	nd	+
RORβ	+	–	–	–	–	+	–	nd	–
RORα	–	+	–	–	–	+	nd	nd	nd
Lbx1	Nd	nd	nd	nd	nd	+	+	+	nd
MafA	–	–	–	–	–	“+”	+	+	–
MafB	–	+	+	–	nd	“+”	“+”	nd	–
c-Maf	–	–	–	“+”	nd	+	+	–	nd

The first five columns (1–5) refer to inhibitory neuron cell types, while the last four columns (6–9) denote excitatory neurons. The different markers that were analyzed are indicated. The numbering of each column refers to the number assigned to each neuronal population and it is used in all the figures to indicate neurons representative of these columns. Annotation: +, expressed; −, not expressed; nd, not determined; “+”, inferred by indirect evidence. Fuller descriptions of the indirect evidence used for expression profiles 4,6 and 7 are provided in the results section. Asterisk indicates *RORα* ^Cre^; *R26* ^floxstop-Tomato^ expression.

**Figure 6:**
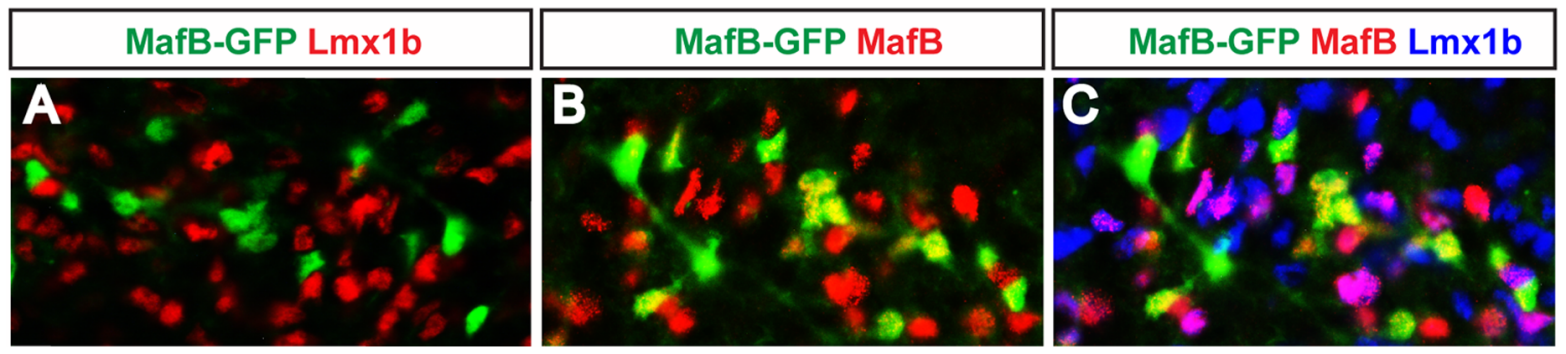
GFP expression in the dorsal horn of *MafB-GFP* mice is restricted to inhibitory neurons. (A) GFP is not expressed in any Lmx1b-positive neurons. (B) Whereas all GFP^+^ neurons are MafB^+^, many MafB neurons do not express GFP. (C) MafB^+^ neurons that are Lmx1b^+^ do not express GFP.

Among the five inhibitory neuron populations, there is one that does not express Gbx1. These cells can be seen in the *GAD67-GFP* spinal cord, where there are a number of GFP^+^ cells in laminae III–IV that do not express Gbx1 (Fig. 2B,B′: Table 2, column 5). The neurons that express Gbx1 can be further subdivided into four populations: one that expresses RORβ (Table 2, column 1); one that expresses RORα and MafB (Table 2, column 2); one that expresses MafB and not RORα or RORβ (Table 2, column 3); and one that expresses c-Maf but did not express RORβ, RORα or MafB (Table 2, column 4). The neurons that co-express Gbx1 and RORβ (Fig. 4G–H) do not express MafB (Fig. 5A), in so far as we could not find RORβ^+^/Gbx1^+^ cells that express GFP in *MafB-GFP* mice (Fig. 5A–A′; Table 2, column 1). It should be noted that the GFP reporter only labels inhibitory MafB^+^ neurons in these mice (Fig. 6; data not shown). This is probably due to the loss of an enhancer element in the *MafB-GFP* knock-in allele that directs GFP expression in excitatory neurons [22], [32]. A number of the MafB^+^ (GFP^+^) neurons in the *MafB-GFP* spinal cord were found to express Gbx1 alone (Fig. 5A–A′: Table 2, column 2–3). Some of these cells also express RORα, as there is small population of RORα^+^ (Tomato^+^)/Gbx1^+^ cells that express MafB in the *RORα-Cre; R26* ^floxstop-Tomato^ mice (Fig. 5B; Table 2, column 2). There is also a population of Gbx1^+^/MafB^+^ neurons in the *RORα-Cre; R26* ^floxstop-Tomato^ mice that do not express RORa (Tomato), which represents the third population of Gbx1-expressing neurons (Fig. 5B; Table 2, column 3). Finally, in *MafB-GFP* reporter mice, there are Gbx1-expressing neurons that do not express MafB (GFP) or RORβ. These Gbx1^+^/MafB^−/^RORβ^−^ cells constitute the fourth population of Gbx1^+^ neurons (blue cells in Fig. 5A; Table 2, column 4). We have also found a significant fraction of Gbx1^+^ cells that express c-Maf (Fig. 5E). These cells are not part of the Gbx1^+^/MafB^+^ or Gbx1^+^/RORβ^+^ populations, as GFP^+^/c-Maf^+^ cells are rarely, if ever, detected in the *MafB-GFP* spinal cord (Fig. 5C and data not shown), and Lmx1b^−/^RORβ^+^ inhibitory neurons do not express c-Maf (Fig. 5D–D″; Table 2, columns 1 and 4).

Four different populations of excitatory neurons were identified in lamina III–IV on the basis of Lmx1b and MafA expression, three that express MafA (Table 2, column 6–8), and one that expresses Lmx1b, but not MafA (Table 2, column 9; Figs. 3K–L and 5K′). Interestingly, all of MafA^+^ neurons express Lbx1 (Fig. 5J), although in some instances only very weakly (data not shown). Within the MafA population, one subpopulation expresses RORβ. The MafA^+^/RORβ^+^ cells make up approximately half of all RORβ^+^ neurons in lamina III–IV (Fig. 5F; Table 2, column 6). Since RORβ is also co-expressed with Tomato in *RORα-Cre; Rosa26* ^floxstop-Tomato^ mice (KG and SB, unpublished observations), we conclude that this subset of MafA^+^ excitatory neurons most likely expresses a combination of both RORα and RORβ (Table 2, column 6). These neurons also express MafB and c-Maf, as all excitatory RORβ^+^/Lmx1b^+^ neurons express c-Maf and MafB (Fig. 5D–D″ and 5H; Table 2, column 6). c-Maf rarely, if ever, co-localizes with GFP in *MafB-GFP* mice where the GFP reporter selectively labels inhibitory neurons (Fig. 5C), leading us to conclude that many of the MafB neurons in lamina III–IV are excitatory c-Maf^+^ interneurons (Table 2, column 7). Our results also demonstrate that there are twice as many MafA^+^/c-Maf^+^ neurons in lamina III–IV as compared to MafA^+^/RORβ^+^ cells. This means that approximately 50% of the MafA^+^/c-Maf^+^ neurons in lamina III–IV are RORβ-negative (Table 2, column 7). Finally, we have found a small population of MafA^+^ neurons in lamina III–IV that do not express c-Maf (Fig. 5I; Table 2, column 8).

## Discussion

In this study, we describe the identification of nine different populations of postnatal neurons that are principally located in laminae III–IV, the main area for processing cutaneous mechanical stimuli in the spinal cord. The classification of sensory interneurons in lamina III and IV was based on a combinatorial transcription factor code comprising of developmental factors known to regulate cell fate specification in the nervous system. Our findings demonstrate an unanticipated level of diversity in the interneuron populations that are located in regions of the spinal cord receiving low-threshold cutaneous mechanosensory inputs. A more limited diversity was suggested by previous electrophysiological studies in vitro [44]–[48]. In characterizing neurons in laminae III–IV by their mechanoreceptive afferent fiber input and their intrinsic discharge properties, Schneider [47], [48] identified four groups of cells: phasic, delayed-firing and tonic, where the tonic population is comprised of two different groups. In a similar manner, Hochman et al. [44] divided laminae III-V neurons into four categories based on their firing properties following intracellular current injection: single spike, phasic firing, repetitive firing, and delayed firing. Morphological studies have also identified differences in terms of cell/dendrite shape and axon morphology (see [49], [50], and references there in). Cajal described several types of neurons in laminae II–III [51]. In general, lamina III–IV contains neurons of varying sizes and shapes: rounded, slightly elongated, or spindle shaped cells. There is a lower density of these cells in lamina IV, which also contains large cells. The principal neurons of the susbstantia gelatinosa are small neurons with short axons, which can be classified as central cells or Golgi type II neurons. In addition, there are two larger cell types, stalked cells with rounded soma, and islet cells. However, many neurons do not fit into these neat morphological categories, with lamina IV containing medium sized neurons and larger pyramidal type neurons [1], [2], [51], [52]. The dorsal horn also contains a number of projection neurons, the most prominent of these being spinothalamic/spinoparabrachial neurons in lamina I, III and IV, and the dorsal spinocerebellar neurons that are localized ventral-medially in Clarke’s column [1], [2], [7], [53]. The Gbx1^+^ and Pax2^+^ inhibitory interneuron cell types that we have identified are unlikely to be projection neurons, as spinal projection neurons are primarily glutamatergic. Moreover, the majority of Lmx1b^+^ and Tlx3^+^ cells in the dorsal horn are likely to be local circuit interneurons [11], [12], [14].

More recently, multiple subpopulations of dorsal horn glutamatergic and GABAergic neurons have been identified that express various neuropeptides and calcium binding proteins [29], [54]–[57]. Subsets of GABAergic neurons express the Ca2^+^ binding protein parvalbumin, as well as the neuropeptide transmitters neuropeptide Y (NPY), enkephalin, galanin, glycine and thyrotropin-releasing hormone. There are also small populations of GABAergic neurons that express choline acetyltransferase (ChAT) or nitric oxide synthase (NOS). Glutamatergic interneurons express cholescystokinin (CCK), somatostatin and neurotensin [29], [54]–[57]. There is also a subset of enkephalin- positive neurons that are also glutamatergic. The correlation between classifying neurons according to their specific transcription factor profiles (this study) and cell types that have been subdivided according to their morphology or neurotransmitter/electrophysiological properties remains to be determined. Defining these relationships would go a long way toward identifying the functional elements of mechanosensory circuitry in the dorsal horn.

Interestingly, most of the transcription factors that are expressed in laminae III–IV do not label a single or homogeneous population of neurons. Instead, Lbx1, RORβ, MafB and c-Maf are expressed in both excitatory and inhibitory neurons. To date, MafA is the only marker that is restricted to excitatory neurons, and even then, it is expressed in three molecularly-distinct populations of excitatory neurons. Somewhat surprisingly, Hu et al. [58] have reported that MafA largely co-localizes with Pax2, whereas our data show that MafA is a specific marker of postnatal excitatory neurons. MafA does not co-localize with GFP in *GAD67-GFP* mice or with Gbx1 at any of the postnatal stages we investigated. Furthermore, MafA is completely lost in *Lmx1b* mutant mice that express a normal complement of inhibitory gene markers, including Pax2 (Fig. S4). This finding coupled with the observation that MafA is not reduced in *Ptf1a* mutant mice [58], when Ptf1a is known to be required for GABAergic neuron differentiation [29], [59], strongly argues against the expression of MafA in laminae III–IV inhibitory neurons.

While many of the transcription factors analyzed in this study are restricted to the dorsal horn at postnatal times, their expression patterns in the embryonic cord are often broader and encompass neurons that settle in the intermediate and ventral regions of the spinal cord [60]–[64]. The one exception is RORβ. RORβ is expressed in the dorsal horn throughout embryogenesis (Fig. 1; MDB and MG, unpublished data). MafB, for example, is expressed in differentiating Renshaw cells that are derived from ventral p1 progenitors [32]. MafB is also expressed in motor neurons [32]. c-Maf is expressed at E12.5 in dI1 and dI3 neurons in the dorsal horn [31], which are glutamatergic projection neurons that migrate and settle at more ventral locales in the spinal cord. Taken together, these data make it highly unlikely that any single transcription factor specifies cell type in the dorsal horn. They instead point to neuronal cell identity in the dorsal spinal cord being determined by the combinatorial activities of multiple transcription factors.

Although Lbx1, Lmx1b and Tlx3 transcription factors all have essential roles in neuronal specification and differentiation during the two waves of neurogenesis that give rise to dorsal horn interneurons [11], [12], [14], [15], they continue to be expressed in subsets of lamina III–IV neurons in the adult when neural differentiation has ceased. The functional importance of this persistent expression is not known. One possibility is that these transcription factors are important for maintaining the identity and mature phenotype of sensory interneurons. For example, Lhx1 and Lhx5 are required to maintain Pax2 expression in mature GABAergic neurons [28]. The maintenance of these factors along with Gbx1, MafB, MafA and c-Maf may also be important for the reorganization of cutaneous sensory afferent inputs to the dorsal horn that occurs during the early postnatal period [3], [65]. For example, Lmx1b is known to play a role in motor neuron axon guidance [66], [67], and it might similarly control axon guidance and remodeling in the dorsal horn. In summary, this study defines a novel transcription factor code for sensory interneurons in lamina III–IV. These first order sensory neurons are the targets of low-threshold cutaneous mechanoreceptors, and their characterization provides a foundation for future experiments to determine how sensory neurons in the dorsal horn encode cutaneous tactile information.

## Supporting Information

Figure S1 **MafA, MafB and c-Maf label different populations of intermedial and ventral neurons.** At E11, MafA (A–C), MafB (D–F) and c-Maf (G–H) label different populations of neurons that distinct from laminae III–IV neurons due to their relative expression of Lmx1b and Lhx1/5. At E13, MafB (G) and c-Maf (H) label a population of neurons that are not in laminae III–IV due to their position relative to Lmx1b.
(TIF)

Figure S2 **Lbx1, MafA, Gbx1, RORβ and c-Maf are markers of postnatal mechanosensory interneurons in laminae III–IV.** VGluT1 labels mechanosensory afferents that terminate mainly in inner lamina II - dorsal lamina V. The neurons expressing the Lbx1, Gbx1, RORb, MafA and c-Maf transcription factors are located in laminae III–IV at early postnatal (A–C), late postnatal (D) and young adult stages (E).
(TIF)

Figure S3 ***Pax2-Cre*** **recombines reporter expression in Pax2^+^ inhibitory neurons.** Comparative expression of Pax2 and Lmx1b following *Pax2-Cre*-mediated recombination. Note the near complete overlap in Pax2 and nuclear GFP expression at E16.5 (A–B), whereas nuclear GFP expression is completely excluded from Lmx1b^+^ excitatory neurons (C–D).
(TIF)

Figure S4 **MafA expression in the dorsal horn of** ***Lmx1b*** **mutant mice.** Control (C1 and C2) and *Lmx1b* mutant animals (M1 and M2) were analyzed at P0. Lmx1b is expressed in control mice (A and B), but not in *Lmx1b* mutant mice (C and D). Expression of MafA in the dorsal spinal cord (E and F) is is also lost in the *Lmx1b* mutant mice (G and H). Pax2 expression in inhibitory neurons is maintained in *Lmx1b* mutant mice (K and L) in a pattern that is comparable to control mice (I and J).
(TIF)
