# Supplementary figures and images for: A Transcription Factor Code Defines Nine Sensory Interneuron Subtypes in the Mechanosensory Area of the Spinal Cord
Source: PLoS One. 2013 Nov 4;8(11):e77928. doi: 10.1371/journal.pone.0077928 (PMC3817166; doi:10.1371/journal.pone.0077928)

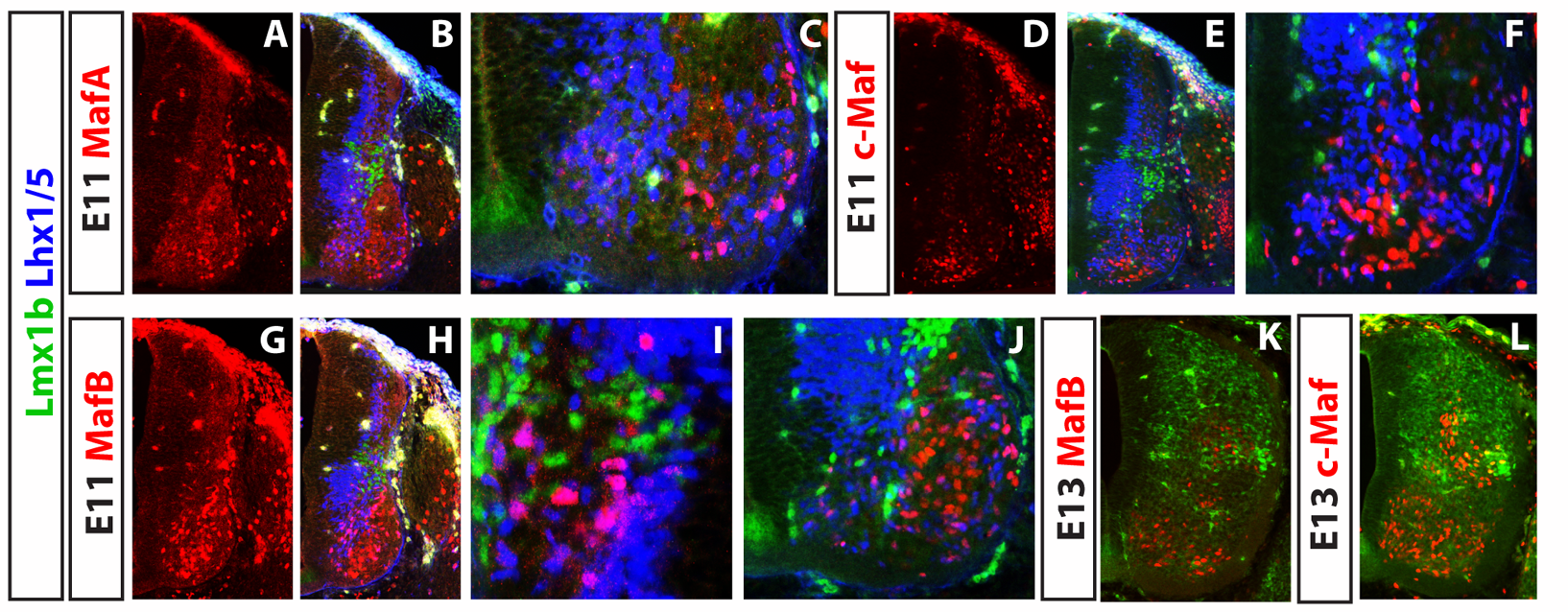

Supplement: Figure S1 — MafA, MafB and c-Maf label different populations of intermedial and ventral neurons. At E11, MafA (A–C), MafB (D–F) and c-Maf (G–H) label different populations of neurons that distinct from laminae III–IV neurons due to their relative expression of Lmx1b and Lhx1/5. At E13, MafB (G) and c-Maf (H) label a population of neurons that are not in laminae III–IV due to their position relative to Lmx1b. (TIF) [file pone.0077928.s001.tif]

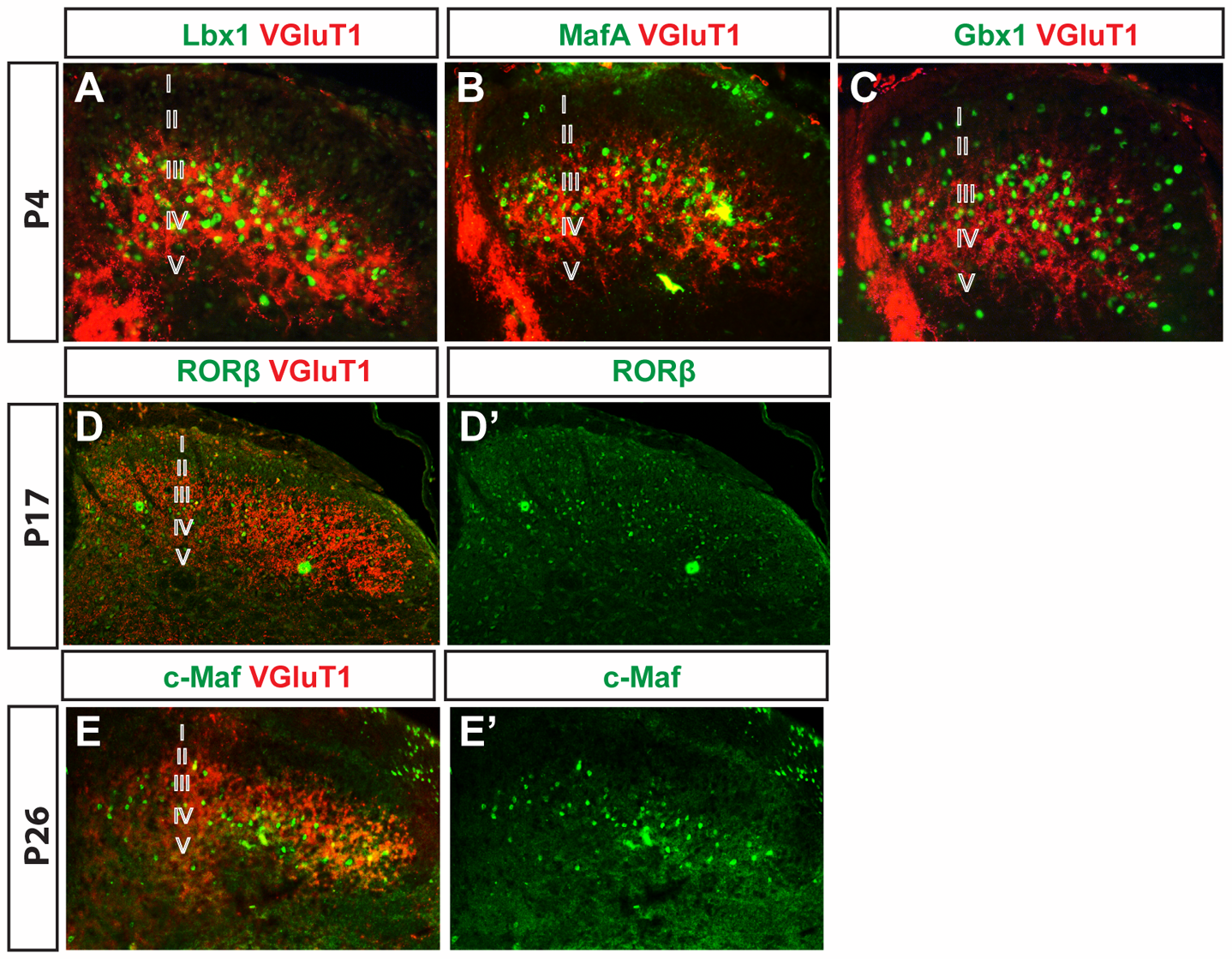

Supplement: Figure S2 — Lbx1, MafA, Gbx1, RORβ and c-Maf are markers of postnatal mechanosensory interneurons in laminae III–IV. VGluT1 labels mechanosensory afferents that terminate mainly in inner lamina II - dorsal lamina V. The neurons expressing the Lbx1, Gbx1, RORb, MafA and c-Maf transcription factors are located in laminae III–IV at early postnatal (A–C), late postnatal (D) and young adult stages (E). (TIF) [file pone.0077928.s002.tif]

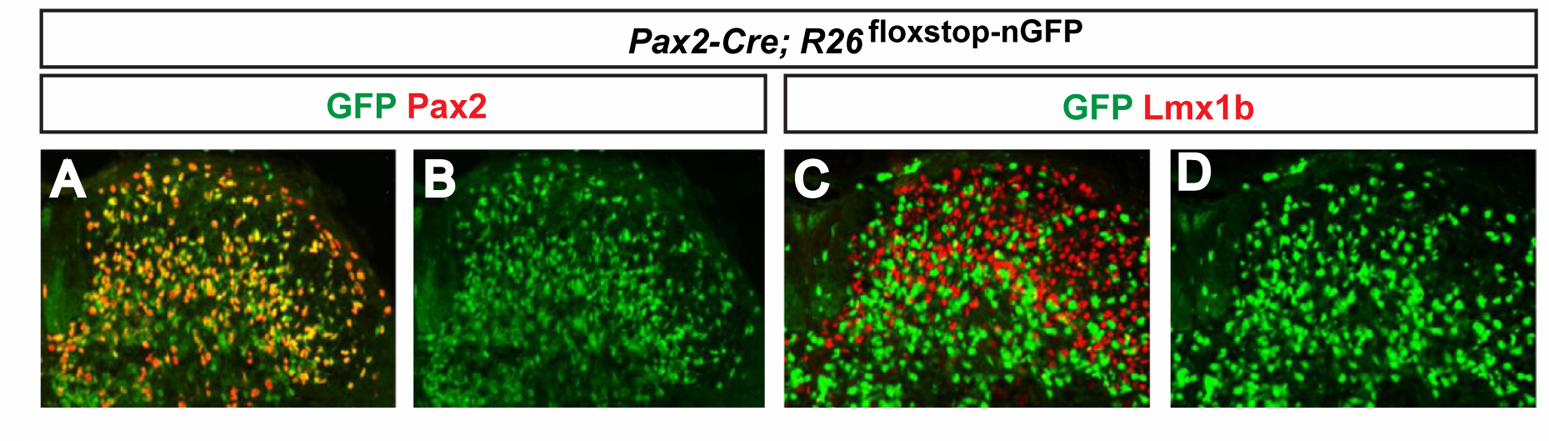

Supplement: Figure S3 — Pax2-Cre recombines reporter expression in Pax2+ inhibitory neurons. Comparative expression of Pax2 and Lmx1b following Pax2-Cre-mediated recombination. Note the near complete overlap in Pax2 and nuclear GFP expression at E16.5 (A–B), whereas nuclear GFP expression is completely excluded from Lmx1b+ excitatory neurons (C–D). (TIF) [file pone.0077928.s003.tif]

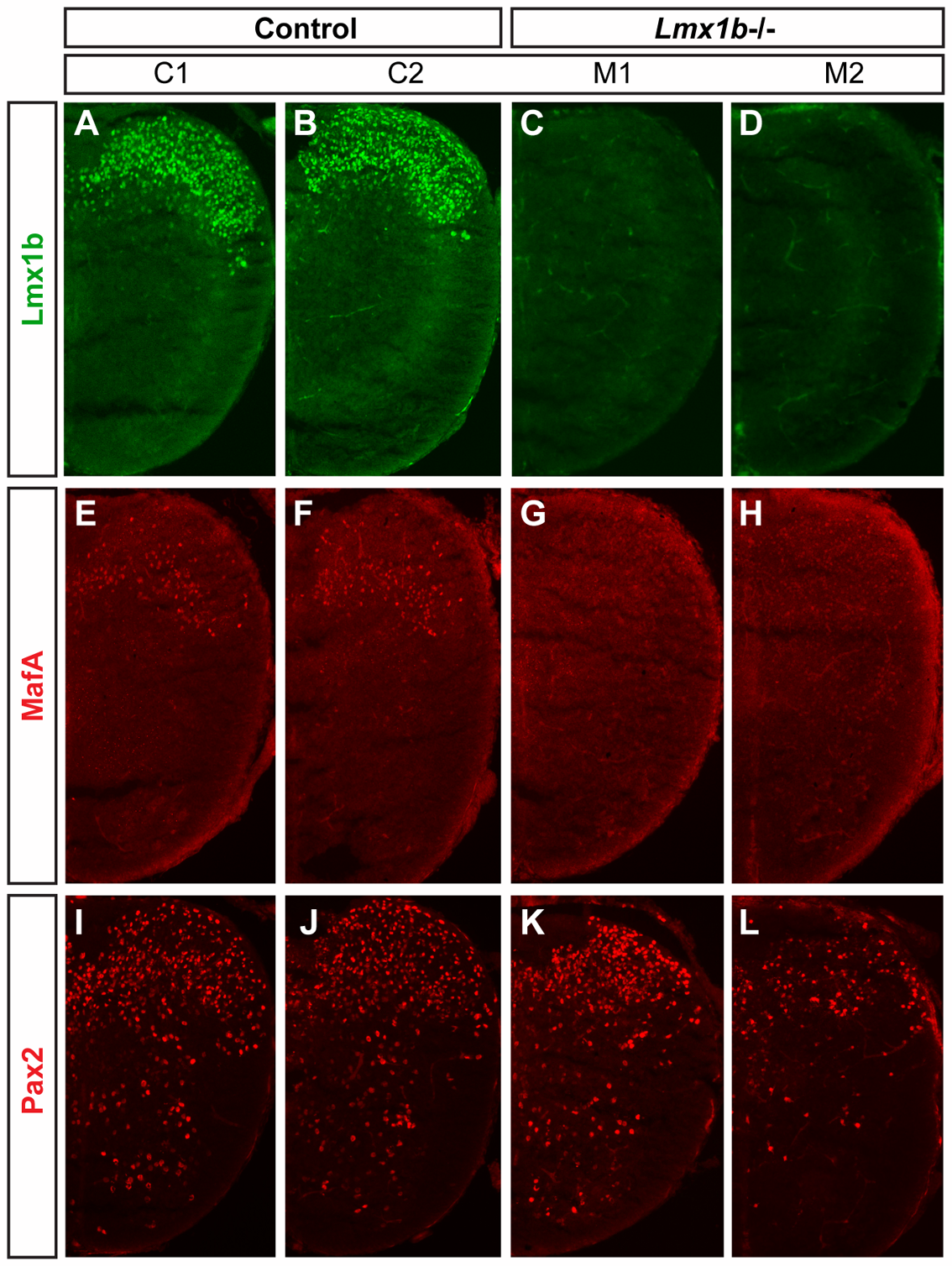

Supplement: Figure S4 — MafA expression in the dorsal horn of Lmx1b mutant mice. Control (C1 and C2) and Lmx1b mutant animals (M1 and M2) were analyzed at P0. Lmx1b is expressed in control mice (A and B), but not in Lmx1b mutant mice (C and D). Expression of MafA in the dorsal spinal cord (E and F) is is also lost in the Lmx1b mutant mice (G and H). Pax2 expression in inhibitory neurons is maintained in Lmx1b mutant mice (K and L) in a pattern that is comparable to control mice (I and J). (TIF) [file pone.0077928.s004.tif]
